# (*E*)-3-(2-Chloro-3,3,3-trifluoro­prop-1-en­yl)-2,2-dimethyl-*N*-(2-naphth­yl)cyclo­propane­carboxamide

**DOI:** 10.1107/S1600536808032078

**Published:** 2008-10-25

**Authors:** Dong-Qing Liu, Fan-Yong Yan

**Affiliations:** a School of Materials and Chemical Engineering, Tianjin Polytechnic University, Tianjin 300160, People’s Republic of China

## Abstract

The title compound, C_19_H_17_ClF_3_NO, was synthesized from 3-[(*E*)-2-chloro-3,3,3-trifluoro­prop-1-en­yl]-2,2-dimethyl­cyclopropane­carboxylic acid and 2-aminona­phthalene. There are two molecules in the asymmetric unit. The dihedral angle between the naphthalene and cyclo­propane units is 111.6 (5)°. Molecules are connected into chains by intermol­ecular N—H⋯O hydrogen bonds.  One of the Cl atoms is disordered over two positions with occupancies 0.653 (15) and 0.347 (15).

## Related literature

For general background, see: Punja (1981 ▶). For synthetic details, see: Liu & Yan (2007 ▶).
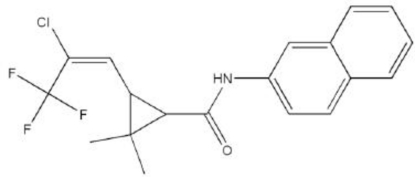


## Experimental

### 

#### Crystal data

C_19_H_17_ClF_3_NO
*M* *_r_* = 367.79
Orthorhombic, 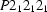

*a* = 9.6310 (8) Å
*b* = 16.9090 (16) Å
*c* = 22.485 (2) Å
*V* = 3661.6 (6) Å^3^
*Z* = 8
Mo *K*α radiation
μ = 0.24 mm^−1^
*T* = 113 (2) K
0.32 × 0.22 × 0.14 mm

#### Data collection

Rigaku Saturn diffractometer
Absorption correction: multi-scan (*CrystalClear*; Rigaku/MSC, 2005 ▶) *T*_min_ = 0.926, *T*_max_ = 0.967
34375 measured reflections
8701 independent reflections
8283 reflections with *I* > 2σ(*I*)
*R*_int_ = 0.046

#### Refinement

*R*[*F* ^2^ > 2σ(*F* ^2^)] = 0.053
*wR*(*F* ^2^) = 0.115
*S* = 1.14
8701 reflections
473 parameters
H atoms treated by a mixture of independent and constrained refinement
Δρ_max_ = 0.23 e Å^−3^
Δρ_min_ = −0.28 e Å^−3^
Absolute structure: Flack (1983 ▶), 3846 Friedel pairs
Flack parameter: −0.05 (6)

### 

Data collection: *CrystalClear* (Rigaku/MSC, 2005 ▶); cell refinement: *CrystalClear*; data reduction: *CrystalClear*; program(s) used to solve structure: *SHELXS97* (Sheldrick, 2008 ▶); program(s) used to refine structure: *SHELXL97* (Sheldrick, 2008 ▶); molecular graphics: *SHELXTL* (Bruker, 1997 ▶); software used to prepare material for publication: *CrystalStructure* (Rigaku/MSC, 2005 ▶).

## Supplementary Material

Crystal structure: contains datablocks I, global. DOI: 10.1107/S1600536808032078/bt2796sup1.cif

Structure factors: contains datablocks I. DOI: 10.1107/S1600536808032078/bt2796Isup2.hkl

Additional supplementary materials:  crystallographic information; 3D view; checkCIF report

## Figures and Tables

**Table 1:** Hydrogen-bond geometry (Å, °)

*D*—H⋯*A*	*D*—H	H⋯*A*	*D*⋯*A*	*D*—H⋯*A*
N1—H1⋯O2	0.86 (3)	2.09 (3)	2.932 (2)	167 (2)
N2—H2*A*⋯O1^i^	0.82 (2)	2.19 (3)	3.003 (2)	169 (2)

Symmetry code: (i) 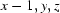.

**Fig. 1.**
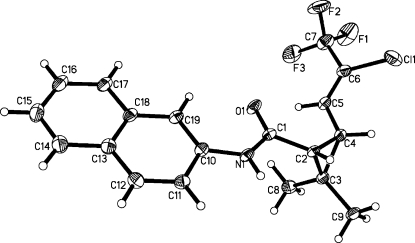
The molecular structure of (I), methanol, drawn with 30% probability ellipsoids. H atoms are drawn as spheres of arbitrary radius.

**Fig. 2.**
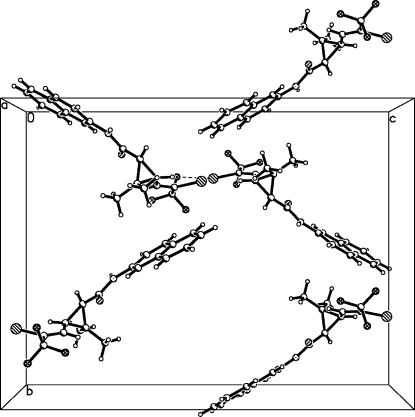
The crystal structure of (I), methanol, viewed along b axis

## supplementary crystallographic information

### Comment

3-[(*E*)-2-Chloro-3,3,3-trifluoroprop-1-enyl]-2,2-dimethylcyclopropanecarboxylic acid is a very important intermediate for tefluthrin, an important insecticide controlling a wide range of soil insect pests in maize,sugar beet, and other crops (Punja, 1981). Naphthalene is also a good structure which has bioactivity. The structure in this article containing both of two active parts may be show some insecticide activity probably. The present X-ray crystal structure analysis was undertaken in order to study the stereochemistry and crystal packing of the title compound, (I) In this paper, the title compound, (*E*)-3-(2-Chloro-3,3,3-trifluoroprop-1-enyl)-2,2-dimethyl-N-(naphthalen-2-yl)cyclopropanecarboxamide, (I), was synthesized and the structure of (I) was illustrated in Fig. 1. The dihedral angles between the naphthalene moiety and the cycloprapane group is 111.6 (5)°. The amide hydrogen is linking with the amide oxygen in another molecule by an intermolecular N—H···O···H—C hydrogen bond. The packing can be described as a dimeric arrangement of molecules linked through N—H···O···H—C hydrogen bond as shown in Fig. 2 and Table 1.

### Experimental

The title compound was prepared according to the method of Liu & Yan (2007). The product was recrystallized from methanol and ethyl acetate (10:1) over 2 d at ambient temperature, gave colourless single crystals of (*E*)-3-(2-Chloro-3,3,3-trifluoroprop-1-enyl)-2,2-dimethyl-N- (naphthalen-2-yl)cyclopropanecarboxamide, suitable for X-ray analysis.

### Refinement

H atoms were positioned geometrically with C—H = 0.93–0.98 Å and refined using riding model with *U*_iso_(H) = 1.2Ueq(carrier). H atom of N—H was located from difference map and refinded freely.

### Crystal data

**Table:**

C_19_H_17_ClF_3_NO	*D*_x_ = 1.334 Mg m^−^^3^
*M**_r_* = 367.79	Mo *K*α radiation, λ = 0.71070 Å
Orthorhombic, *P*2_1_2_1_2_1_	Cell parameters from 9431 reflections
*a* = 9.6310 (8) Å	θ = 1.2–27.9°
*b* = 16.9090 (16) Å	µ = 0.24 mm^−^^1^
*c* = 22.485 (2) Å	*T* = 113 K
*V* = 3661.6 (6) Å^3^	Block, colourless
*Z* = 8	0.32 × 0.22 × 0.14 mm
*F*(000) = 1520	

### Data collection

**Table:**

Rigaku Saturn diffractometer	8701 independent reflections
Radiation source: rotating anode	8283 reflections with *I* > 2σ(*I*)
confocal	*R*_int_ = 0.046
Detector resolution: 14.63 pixels mm^-1^	θ_max_ = 27.9°, θ_min_ = 1.5°
ω scans	*h* = −12→12
Absorption correction: multi-scan (CrystalClear; Rigaku/MSC, 2005)	*k* = −22→21
*T*_min_ = 0.926, *T*_max_ = 0.967	*l* = −29→29
34375 measured reflections	

### Refinement

**Table:**

Refinement on *F*^2^	Secondary atom site location: difference Fourier map
Least-squares matrix: full	Hydrogen site location: inferred from neighbouring sites
*R*[*F*^2^ > 2σ(*F*^2^)] = 0.053	H atoms treated by a mixture of independent and constrained refinement
*wR*(*F*^2^) = 0.115	*w* = 1/[σ^2^(*F*_o_^2^) + (0.0431*P*)^2^ + 0.7651*P*] where *P* = (*F*_o_^2^ + 2*F*_c_^2^)/3
*S* = 1.14	(Δ/σ)_max_ = 0.004
8701 reflections	Δρ_max_ = 0.23 e Å^−^^3^
473 parameters	Δρ_min_ = −0.28 e Å^−^^3^
0 restraints	Absolute structure: Flack (1983), with how many Friedel pairs?
Primary atom site location: structure-invariant direct methods	Flack parameter: −0.05 (6)

### Special details

**Table:**

Geometry. All e.s.d.'s (except the e.s.d. in the dihedral angle between two l.s. planes) are estimated using the full covariance matrix. The cell e.s.d.'s are taken into account individually in the estimation of e.s.d.'s in distances, angles and torsion angles; correlations between e.s.d.'s in cell parameters are only used when they are defined by crystal symmetry. An approximate (isotropic) treatment of cell e.s.d.'s is used for estimating e.s.d.'s involving l.s. planes.
Refinement. Refinement of *F*^2^ against ALL reflections. The weighted *R*-factor *wR* and goodness of fit *S* are based on *F*^2^, conventional *R*-factors *R* are based on *F*, with *F* set to zero for negative *F*^2^. The threshold expression of *F*^2^ > σ(*F*^2^) is used only for calculating *R*-factors(gt) *etc*. and is not relevant to the choice of reflections for refinement. *R*-factors based on *F*^2^ are statistically about twice as large as those based on *F*, and *R*- factors based on ALL data will be even larger.

### Fractional atomic coordinates and isotropic or equivalent isotropic displacement parameters (Å^2^)

**Table:**

	*x*	*y*	*z*	*U*_iso_*/*U*_eq_	Occ. (<1)
Cl1	0.67199 (8)	0.75840 (5)	0.01612 (3)	0.05570 (19)	
Cl2	0.2270 (3)	0.5061 (5)	0.02545 (13)	0.0676 (12)	0.653 (15)
Cl2'	0.2403 (3)	0.5502 (9)	0.0412 (4)	0.070 (3)	0.347 (15)
F1	0.9051 (2)	0.85834 (13)	0.05947 (11)	0.0915 (7)	
F2	0.94983 (18)	0.73920 (13)	0.08421 (10)	0.0805 (6)	
F3	0.89532 (17)	0.82357 (13)	0.15124 (9)	0.0722 (5)	
F4	0.43674 (15)	0.43635 (10)	0.16241 (7)	0.0552 (4)	
F5	0.50212 (16)	0.51978 (12)	0.09630 (9)	0.0693 (5)	
F6	0.44470 (19)	0.40315 (14)	0.07147 (9)	0.0872 (7)	
O1	0.64390 (15)	0.66486 (10)	0.22636 (7)	0.0378 (4)	
O2	0.14813 (15)	0.59263 (9)	0.24189 (7)	0.0329 (3)	
N1	0.45095 (19)	0.59688 (11)	0.25494 (8)	0.0291 (4)	
N2	−0.05216 (19)	0.66161 (11)	0.25605 (8)	0.0283 (4)	
C1	0.5205 (2)	0.64882 (12)	0.21976 (10)	0.0277 (4)	
C2	0.4298 (2)	0.68560 (12)	0.17359 (9)	0.0264 (4)	
H2	0.3594	0.6491	0.1558	0.032*	
C3	0.3824 (2)	0.77078 (13)	0.17939 (9)	0.0304 (5)	
C4	0.4844 (2)	0.74882 (13)	0.13117 (9)	0.0302 (5)	
H4	0.4443	0.7468	0.0902	0.036*	
C5	0.6308 (2)	0.77356 (13)	0.13406 (10)	0.0324 (5)	
H5	0.6664	0.7866	0.1722	0.039*	
C6	0.7173 (2)	0.77930 (14)	0.08877 (11)	0.0362 (5)	
C7	0.8664 (3)	0.80168 (18)	0.09634 (14)	0.0542 (7)	
C8	0.4284 (3)	0.81968 (14)	0.23245 (11)	0.0390 (5)	
H8A	0.5227	0.8042	0.2440	0.059*	
H8B	0.3649	0.8105	0.2658	0.059*	
H8C	0.4274	0.8759	0.2218	0.059*	
C9	0.2351 (2)	0.78755 (15)	0.15899 (11)	0.0387 (5)	
H9A	0.2280	0.8427	0.1460	0.058*	
H9B	0.1707	0.7783	0.1920	0.058*	
H9C	0.2116	0.7524	0.1258	0.058*	
C10	0.5010 (2)	0.55608 (12)	0.30557 (9)	0.0283 (4)	
C11	0.4000 (2)	0.51516 (13)	0.33956 (10)	0.0322 (5)	
H11	0.3058	0.5150	0.3270	0.039*	
C12	0.4372 (3)	0.47600 (14)	0.39024 (10)	0.0358 (5)	
H12	0.3683	0.4484	0.4123	0.043*	
C13	0.5761 (3)	0.47564 (13)	0.41061 (10)	0.0343 (5)	
C14	0.6182 (3)	0.43805 (14)	0.46424 (11)	0.0431 (6)	
H14	0.5521	0.4097	0.4873	0.052*	
C15	0.7531 (3)	0.44237 (15)	0.48295 (12)	0.0470 (6)	
H15	0.7797	0.4176	0.5191	0.056*	
C16	0.8524 (3)	0.48304 (15)	0.44914 (11)	0.0443 (6)	
H16	0.9456	0.4859	0.4628	0.053*	
C17	0.8165 (2)	0.51854 (14)	0.39682 (10)	0.0374 (5)	
H17	0.8854	0.5449	0.3740	0.045*	
C18	0.6773 (2)	0.51631 (13)	0.37624 (10)	0.0312 (4)	
C19	0.6375 (2)	0.55512 (12)	0.32312 (9)	0.0299 (4)	
H19	0.7058	0.5807	0.2994	0.036*	
C20	0.0286 (2)	0.60868 (12)	0.22699 (9)	0.0254 (4)	
C21	−0.0453 (2)	0.56913 (13)	0.17712 (9)	0.0277 (4)	
H21	−0.1168	0.6026	0.1570	0.033*	
C22	−0.0824 (2)	0.48145 (13)	0.18118 (9)	0.0289 (4)	
C23	0.0254 (2)	0.50805 (13)	0.13649 (9)	0.0288 (4)	
H23	−0.0081	0.5088	0.0944	0.035*	
C24	0.1721 (2)	0.48659 (13)	0.14322 (9)	0.0298 (4)	
H24	0.2022	0.4704	0.1816	0.036*	
C25	0.2655 (2)	0.48783 (16)	0.10067 (10)	0.0391 (5)	
C26	0.4110 (3)	0.46160 (17)	0.10780 (12)	0.0463 (6)	
C27	−0.0401 (2)	0.43473 (14)	0.23534 (10)	0.0355 (5)	
H27A	−0.1138	0.4377	0.2653	0.053*	
H27B	0.0459	0.4568	0.2517	0.053*	
H27C	−0.0249	0.3794	0.2241	0.053*	
C28	−0.2230 (2)	0.45972 (15)	0.15708 (11)	0.0395 (5)	
H28A	−0.2247	0.4032	0.1474	0.059*	
H28B	−0.2418	0.4906	0.1211	0.059*	
H28C	−0.2941	0.4711	0.1870	0.059*	
C29	−0.0231 (2)	0.70345 (12)	0.30899 (10)	0.0282 (4)	
C30	−0.1384 (2)	0.74173 (12)	0.33609 (10)	0.0333 (5)	
H30	−0.2276	0.7384	0.3182	0.040*	
C31	−0.1216 (3)	0.78325 (14)	0.38760 (11)	0.0384 (5)	
H31	−0.1997	0.8083	0.4052	0.046*	
C32	0.0095 (3)	0.78970 (14)	0.41532 (11)	0.0384 (5)	
C33	0.0309 (3)	0.83374 (15)	0.46851 (12)	0.0491 (7)	
H33	−0.0450	0.8605	0.4865	0.059*	
C34	0.1589 (3)	0.83780 (15)	0.49368 (11)	0.0514 (7)	
H34	0.1715	0.8671	0.5293	0.062*	
C35	0.2722 (3)	0.79948 (15)	0.46779 (11)	0.0479 (7)	
H35	0.3611	0.8032	0.4859	0.057*	
C36	0.2568 (3)	0.75658 (14)	0.41665 (11)	0.0409 (6)	
H36	0.3347	0.7305	0.3997	0.049*	
C37	0.1248 (2)	0.75090 (13)	0.38880 (10)	0.0338 (5)	
C38	0.1057 (2)	0.70761 (12)	0.33494 (10)	0.0312 (5)	
H38	0.1824	0.6816	0.3170	0.037*	
H2A	−0.132 (3)	0.6668 (13)	0.2440 (9)	0.022 (6)*	
H1	0.365 (3)	0.5892 (15)	0.2472 (11)	0.039 (7)*	

### Atomic displacement parameters (Å^2^)

**Table:**

	*U*^11^	*U*^22^	*U*^33^	*U*^12^	*U*^13^	*U*^23^
Cl1	0.0553 (4)	0.0665 (4)	0.0453 (3)	−0.0042 (3)	0.0152 (3)	−0.0084 (3)
Cl2	0.0340 (7)	0.136 (3)	0.0327 (7)	0.0050 (11)	0.0050 (5)	0.0149 (14)
Cl2'	0.0385 (13)	0.131 (6)	0.040 (3)	−0.0058 (19)	−0.0029 (12)	0.039 (3)
F1	0.0505 (11)	0.0977 (14)	0.1265 (18)	−0.0318 (11)	−0.0061 (12)	0.0623 (14)
F2	0.0335 (9)	0.0963 (15)	0.1117 (16)	0.0213 (10)	0.0170 (10)	0.0165 (13)
F3	0.0329 (9)	0.0979 (14)	0.0857 (13)	−0.0179 (9)	−0.0102 (9)	0.0028 (11)
F4	0.0276 (7)	0.0795 (11)	0.0584 (9)	0.0131 (7)	−0.0018 (7)	0.0173 (9)
F5	0.0238 (8)	0.0884 (13)	0.0956 (14)	−0.0049 (8)	0.0092 (8)	0.0256 (11)
F6	0.0486 (11)	0.1165 (17)	0.0963 (15)	0.0210 (11)	0.0111 (10)	−0.0522 (13)
O1	0.0180 (7)	0.0449 (9)	0.0506 (9)	−0.0053 (7)	−0.0075 (7)	0.0136 (8)
O2	0.0172 (7)	0.0385 (8)	0.0431 (9)	0.0025 (6)	−0.0058 (6)	−0.0056 (7)
N1	0.0168 (8)	0.0326 (9)	0.0380 (10)	−0.0030 (7)	−0.0057 (8)	0.0016 (8)
N2	0.0169 (8)	0.0321 (9)	0.0359 (10)	0.0001 (7)	−0.0042 (7)	−0.0027 (8)
C1	0.0196 (10)	0.0286 (10)	0.0351 (11)	0.0003 (8)	−0.0020 (8)	−0.0005 (9)
C2	0.0166 (9)	0.0311 (10)	0.0315 (11)	−0.0012 (8)	−0.0027 (8)	−0.0002 (8)
C3	0.0241 (10)	0.0331 (11)	0.0338 (11)	0.0020 (9)	−0.0005 (9)	0.0052 (9)
C4	0.0232 (10)	0.0389 (12)	0.0283 (10)	−0.0005 (9)	−0.0021 (8)	0.0029 (9)
C5	0.0251 (11)	0.0345 (11)	0.0375 (11)	0.0006 (9)	−0.0051 (9)	0.0047 (9)
C6	0.0268 (11)	0.0375 (12)	0.0443 (13)	0.0023 (9)	0.0036 (10)	0.0052 (10)
C7	0.0314 (14)	0.0605 (17)	0.0706 (19)	−0.0041 (13)	0.0048 (13)	0.0268 (15)
C8	0.0375 (13)	0.0362 (12)	0.0434 (13)	0.0041 (10)	0.0015 (10)	−0.0019 (10)
C9	0.0244 (11)	0.0462 (13)	0.0455 (13)	0.0066 (10)	0.0006 (10)	0.0105 (11)
C10	0.0256 (10)	0.0256 (10)	0.0336 (11)	−0.0017 (8)	−0.0030 (8)	0.0002 (8)
C11	0.0264 (10)	0.0332 (11)	0.0372 (11)	−0.0040 (9)	−0.0008 (9)	−0.0022 (9)
C12	0.0372 (13)	0.0338 (11)	0.0365 (12)	−0.0045 (10)	0.0023 (10)	−0.0004 (10)
C13	0.0405 (13)	0.0285 (10)	0.0337 (11)	0.0042 (10)	−0.0016 (10)	−0.0047 (9)
C14	0.0549 (16)	0.0376 (12)	0.0369 (12)	0.0071 (12)	−0.0002 (11)	−0.0007 (10)
C15	0.0618 (18)	0.0434 (14)	0.0359 (12)	0.0157 (13)	−0.0104 (12)	−0.0018 (11)
C16	0.0430 (14)	0.0435 (13)	0.0465 (13)	0.0150 (12)	−0.0140 (12)	−0.0067 (11)
C17	0.0328 (12)	0.0372 (11)	0.0421 (12)	0.0097 (10)	−0.0086 (10)	−0.0051 (10)
C18	0.0300 (11)	0.0275 (10)	0.0360 (11)	0.0062 (9)	−0.0050 (9)	−0.0051 (9)
C19	0.0233 (10)	0.0292 (10)	0.0372 (11)	0.0000 (8)	−0.0023 (9)	0.0001 (9)
C20	0.0193 (10)	0.0244 (9)	0.0324 (11)	−0.0038 (8)	−0.0019 (8)	0.0028 (8)
C21	0.0168 (9)	0.0337 (10)	0.0327 (11)	0.0026 (8)	−0.0031 (8)	0.0011 (9)
C22	0.0195 (10)	0.0333 (10)	0.0340 (11)	−0.0045 (8)	0.0011 (8)	−0.0026 (9)
C23	0.0203 (10)	0.0375 (11)	0.0288 (10)	−0.0013 (9)	−0.0019 (8)	−0.0043 (9)
C24	0.0228 (10)	0.0358 (11)	0.0309 (10)	−0.0016 (9)	−0.0019 (8)	−0.0010 (9)
C25	0.0253 (11)	0.0556 (15)	0.0365 (12)	0.0023 (11)	0.0034 (9)	0.0058 (11)
C26	0.0276 (12)	0.0649 (17)	0.0463 (14)	0.0038 (12)	0.0086 (11)	−0.0043 (13)
C27	0.0314 (12)	0.0349 (11)	0.0401 (12)	−0.0040 (10)	0.0051 (10)	0.0034 (10)
C28	0.0231 (11)	0.0473 (13)	0.0480 (13)	−0.0086 (10)	−0.0025 (10)	−0.0111 (11)
C29	0.0255 (10)	0.0261 (10)	0.0331 (11)	−0.0047 (8)	−0.0008 (9)	−0.0004 (8)
C30	0.0266 (11)	0.0290 (10)	0.0444 (12)	−0.0013 (9)	0.0028 (9)	−0.0007 (9)
C31	0.0342 (13)	0.0323 (11)	0.0488 (14)	−0.0008 (10)	0.0067 (11)	−0.0032 (10)
C32	0.0468 (14)	0.0323 (11)	0.0360 (12)	−0.0070 (10)	0.0018 (10)	−0.0020 (10)
C33	0.0654 (19)	0.0384 (13)	0.0434 (14)	−0.0113 (13)	0.0072 (13)	−0.0069 (11)
C34	0.075 (2)	0.0415 (14)	0.0380 (13)	−0.0180 (14)	−0.0051 (14)	−0.0040 (11)
C35	0.0603 (18)	0.0410 (13)	0.0422 (14)	−0.0143 (13)	−0.0188 (13)	0.0032 (11)
C36	0.0441 (14)	0.0369 (12)	0.0417 (12)	−0.0079 (11)	−0.0108 (11)	0.0018 (10)
C37	0.0379 (13)	0.0294 (11)	0.0342 (11)	−0.0051 (9)	−0.0049 (10)	0.0027 (9)
C38	0.0253 (10)	0.0304 (10)	0.0379 (12)	−0.0001 (8)	−0.0027 (9)	−0.0026 (9)

### Geometric parameters (Å, °)

**Table:**

Cl1—C6	1.727 (3)	C14—H14	0.9500
Cl2—Cl2'	0.836 (9)	C15—C16	1.402 (4)
Cl2—C25	1.759 (3)	C15—H15	0.9500
Cl2'—C25	1.721 (4)	C16—C17	1.365 (3)
F1—C7	1.321 (3)	C16—H16	0.9500
F2—C7	1.355 (3)	C17—C18	1.419 (3)
F3—C7	1.318 (4)	C17—H17	0.9500
F4—C26	1.324 (3)	C18—C19	1.416 (3)
F5—C26	1.344 (3)	C19—H19	0.9500
F6—C26	1.323 (3)	C20—C21	1.487 (3)
O1—C1	1.228 (2)	C21—C22	1.528 (3)
O2—C20	1.230 (2)	C21—C23	1.538 (3)
N1—C1	1.358 (3)	C21—H21	1.0000
N1—C10	1.416 (3)	C22—C28	1.504 (3)
N1—H1	0.86 (3)	C22—C27	1.508 (3)
N2—C20	1.354 (3)	C22—C23	1.513 (3)
N2—C29	1.413 (3)	C23—C24	1.467 (3)
N2—H2A	0.82 (2)	C23—H23	1.0000
C1—C2	1.492 (3)	C24—C25	1.313 (3)
C2—C3	1.517 (3)	C24—H24	0.9500
C2—C4	1.526 (3)	C25—C26	1.478 (3)
C2—H2	1.0000	C27—H27A	0.9800
C3—C4	1.509 (3)	C27—H27B	0.9800
C3—C8	1.518 (3)	C27—H27C	0.9800
C3—C9	1.518 (3)	C28—H28A	0.9800
C4—C5	1.472 (3)	C28—H28B	0.9800
C4—H4	1.0000	C28—H28C	0.9800
C5—C6	1.319 (3)	C29—C38	1.373 (3)
C5—H5	0.9500	C29—C30	1.422 (3)
C6—C7	1.495 (4)	C30—C31	1.364 (3)
C8—H8A	0.9800	C30—H30	0.9500
C8—H8B	0.9800	C31—C32	1.413 (4)
C8—H8C	0.9800	C31—H31	0.9500
C9—H9A	0.9800	C32—C37	1.421 (3)
C9—H9B	0.9800	C32—C33	1.424 (3)
C9—H9C	0.9800	C33—C34	1.359 (4)
C10—C19	1.373 (3)	C33—H33	0.9500
C10—C11	1.417 (3)	C34—C35	1.397 (4)
C11—C12	1.366 (3)	C34—H34	0.9500
C11—H11	0.9500	C35—C36	1.368 (3)
C12—C13	1.414 (3)	C35—H35	0.9500
C12—H12	0.9500	C36—C37	1.421 (3)
C13—C18	1.421 (3)	C36—H36	0.9500
C13—C14	1.422 (3)	C37—C38	1.427 (3)
C14—C15	1.368 (4)	C38—H38	0.9500
			
Cl2'—Cl2—C25	73.6 (3)	C10—C19—H19	119.8
Cl2—Cl2'—C25	78.6 (5)	C18—C19—H19	119.8
C1—N1—C10	128.00 (18)	O2—C20—N2	123.53 (19)
C1—N1—H1	117.1 (17)	O2—C20—C21	123.67 (19)
C10—N1—H1	114.8 (17)	N2—C20—C21	112.74 (17)
C20—N2—C29	128.63 (18)	C20—C21—C22	120.22 (18)
C20—N2—H2A	116.8 (15)	C20—C21—C23	122.55 (17)
C29—N2—H2A	114.2 (15)	C22—C21—C23	59.16 (14)
O1—C1—N1	123.3 (2)	C20—C21—H21	114.6
O1—C1—C2	123.93 (19)	C22—C21—H21	114.6
N1—C1—C2	112.73 (17)	C23—C21—H21	114.6
C1—C2—C3	120.80 (18)	C28—C22—C27	113.99 (19)
C1—C2—C4	121.70 (17)	C28—C22—C23	116.81 (18)
C3—C2—C4	59.48 (14)	C27—C22—C23	120.43 (18)
C1—C2—H2	114.6	C28—C22—C21	115.22 (19)
C3—C2—H2	114.6	C27—C22—C21	119.57 (18)
C4—C2—H2	114.6	C23—C22—C21	60.75 (14)
C4—C3—C2	60.57 (14)	C24—C23—C22	121.26 (19)
C4—C3—C8	120.59 (19)	C24—C23—C21	122.10 (18)
C2—C3—C8	119.82 (18)	C22—C23—C21	60.09 (13)
C4—C3—C9	115.91 (18)	C24—C23—H23	114.3
C2—C3—C9	115.64 (19)	C22—C23—H23	114.3
C8—C3—C9	114.12 (19)	C21—C23—H23	114.3
C5—C4—C3	121.43 (19)	C25—C24—C23	125.5 (2)
C5—C4—C2	120.08 (18)	C25—C24—H24	117.3
C3—C4—C2	59.95 (14)	C23—C24—H24	117.3
C5—C4—H4	114.8	C24—C25—C26	124.4 (2)
C3—C4—H4	114.8	C24—C25—Cl2'	118.7 (3)
C2—C4—H4	114.8	C26—C25—Cl2'	113.7 (2)
C6—C5—C4	126.3 (2)	C24—C25—Cl2	124.02 (19)
C6—C5—H5	116.9	C26—C25—Cl2	110.87 (18)
C4—C5—H5	116.9	Cl2'—C25—Cl2	27.8 (3)
C5—C6—C7	122.5 (2)	F6—C26—F4	106.6 (2)
C5—C6—Cl1	123.73 (19)	F6—C26—F5	105.5 (2)
C7—C6—Cl1	113.73 (19)	F4—C26—F5	107.0 (2)
F3—C7—F1	108.9 (3)	F6—C26—C25	113.0 (2)
F3—C7—F2	106.4 (2)	F4—C26—C25	112.0 (2)
F1—C7—F2	105.8 (2)	F5—C26—C25	112.3 (2)
F3—C7—C6	112.4 (2)	C22—C27—H27A	109.5
F1—C7—C6	112.5 (2)	C22—C27—H27B	109.5
F2—C7—C6	110.4 (3)	H27A—C27—H27B	109.5
C3—C8—H8A	109.5	C22—C27—H27C	109.5
C3—C8—H8B	109.5	H27A—C27—H27C	109.5
H8A—C8—H8B	109.5	H27B—C27—H27C	109.5
C3—C8—H8C	109.5	C22—C28—H28A	109.5
H8A—C8—H8C	109.5	C22—C28—H28B	109.5
H8B—C8—H8C	109.5	H28A—C28—H28B	109.5
C3—C9—H9A	109.5	C22—C28—H28C	109.5
C3—C9—H9B	109.5	H28A—C28—H28C	109.5
H9A—C9—H9B	109.5	H28B—C28—H28C	109.5
C3—C9—H9C	109.5	C38—C29—N2	124.2 (2)
H9A—C9—H9C	109.5	C38—C29—C30	120.0 (2)
H9B—C9—H9C	109.5	N2—C29—C30	115.76 (19)
C19—C10—N1	124.25 (19)	C31—C30—C29	120.4 (2)
C19—C10—C11	119.77 (19)	C31—C30—H30	119.8
N1—C10—C11	115.98 (19)	C29—C30—H30	119.8
C12—C11—C10	120.4 (2)	C30—C31—C32	121.4 (2)
C12—C11—H11	119.8	C30—C31—H31	119.3
C10—C11—H11	119.8	C32—C31—H31	119.3
C11—C12—C13	121.4 (2)	C31—C32—C37	118.5 (2)
C11—C12—H12	119.3	C31—C32—C33	122.7 (3)
C13—C12—H12	119.3	C37—C32—C33	118.8 (2)
C12—C13—C18	118.1 (2)	C34—C33—C32	120.5 (3)
C12—C13—C14	123.1 (2)	C34—C33—H33	119.7
C18—C13—C14	118.8 (2)	C32—C33—H33	119.7
C15—C14—C13	120.5 (3)	C33—C34—C35	120.8 (2)
C15—C14—H14	119.7	C33—C34—H34	119.6
C13—C14—H14	119.7	C35—C34—H34	119.6
C14—C15—C16	120.5 (2)	C36—C35—C34	120.8 (3)
C14—C15—H15	119.8	C36—C35—H35	119.6
C16—C15—H15	119.8	C34—C35—H35	119.6
C17—C16—C15	120.7 (2)	C35—C36—C37	120.2 (3)
C17—C16—H16	119.7	C35—C36—H36	119.9
C15—C16—H16	119.7	C37—C36—H36	119.9
C16—C17—C18	120.6 (2)	C32—C37—C36	118.9 (2)
C16—C17—H17	119.7	C32—C37—C38	119.5 (2)
C18—C17—H17	119.7	C36—C37—C38	121.6 (2)
C19—C18—C17	121.2 (2)	C29—C38—C37	120.2 (2)
C19—C18—C13	119.8 (2)	C29—C38—H38	119.9
C17—C18—C13	118.9 (2)	C37—C38—H38	119.9
C10—C19—C18	120.5 (2)		
			
C10—N1—C1—O1	3.6 (4)	O2—C20—C21—C23	−4.4 (3)
C10—N1—C1—C2	−174.8 (2)	N2—C20—C21—C23	178.52 (18)
O1—C1—C2—C3	−72.5 (3)	C20—C21—C22—C28	139.9 (2)
N1—C1—C2—C3	106.0 (2)	C23—C21—C22—C28	−107.9 (2)
O1—C1—C2—C4	−1.6 (3)	C20—C21—C22—C27	−1.7 (3)
N1—C1—C2—C4	176.88 (19)	C23—C21—C22—C27	110.5 (2)
C1—C2—C3—C4	111.0 (2)	C20—C21—C22—C23	−112.2 (2)
C1—C2—C3—C8	0.5 (3)	C28—C22—C23—C24	−143.1 (2)
C4—C2—C3—C8	−110.5 (2)	C27—C22—C23—C24	2.5 (3)
C1—C2—C3—C9	−142.4 (2)	C21—C22—C23—C24	111.6 (2)
C4—C2—C3—C9	106.6 (2)	C28—C22—C23—C21	105.3 (2)
C2—C3—C4—C5	−109.0 (2)	C27—C22—C23—C21	−109.1 (2)
C8—C3—C4—C5	0.3 (3)	C20—C21—C23—C24	−1.9 (3)
C9—C3—C4—C5	144.9 (2)	C22—C21—C23—C24	−110.2 (2)
C8—C3—C4—C2	109.3 (2)	C20—C21—C23—C22	108.3 (2)
C9—C3—C4—C2	−106.2 (2)	C22—C23—C24—C25	160.7 (2)
C1—C2—C4—C5	1.6 (3)	C21—C23—C24—C25	−127.2 (3)
C3—C2—C4—C5	111.2 (2)	C23—C24—C25—C26	−176.4 (2)
C1—C2—C4—C3	−109.5 (2)	C23—C24—C25—Cl2'	25.3 (7)
C3—C4—C5—C6	−158.2 (2)	C23—C24—C25—Cl2	−6.8 (5)
C2—C4—C5—C6	130.7 (2)	Cl2—Cl2'—C25—C24	−109.3 (6)
C4—C5—C6—C7	−177.3 (2)	Cl2—Cl2'—C25—C26	90.1 (4)
C4—C5—C6—Cl1	0.2 (4)	Cl2'—Cl2—C25—C24	87.8 (6)
C5—C6—C7—F3	−7.5 (4)	Cl2'—Cl2—C25—C26	−101.4 (4)
Cl1—C6—C7—F3	174.77 (19)	C24—C25—C26—F6	120.5 (3)
C5—C6—C7—F1	−130.9 (3)	Cl2'—C25—C26—F6	−80.2 (7)
Cl1—C6—C7—F1	51.3 (3)	Cl2—C25—C26—F6	−50.2 (4)
C5—C6—C7—F2	111.1 (3)	C24—C25—C26—F4	0.1 (4)
Cl1—C6—C7—F2	−66.6 (3)	Cl2'—C25—C26—F4	159.4 (7)
C1—N1—C10—C19	−8.9 (4)	Cl2—C25—C26—F4	−170.7 (4)
C1—N1—C10—C11	170.2 (2)	C24—C25—C26—F5	−120.3 (3)
C19—C10—C11—C12	1.2 (3)	Cl2'—C25—C26—F5	39.0 (7)
N1—C10—C11—C12	−178.0 (2)	Cl2—C25—C26—F5	68.9 (4)
C10—C11—C12—C13	0.6 (3)	C20—N2—C29—C38	12.2 (3)
C11—C12—C13—C18	−1.0 (3)	C20—N2—C29—C30	−166.7 (2)
C11—C12—C13—C14	177.7 (2)	C38—C29—C30—C31	0.6 (3)
C12—C13—C14—C15	−177.3 (2)	N2—C29—C30—C31	179.5 (2)
C18—C13—C14—C15	1.3 (3)	C29—C30—C31—C32	0.2 (3)
C13—C14—C15—C16	−0.8 (4)	C30—C31—C32—C37	−0.8 (4)
C14—C15—C16—C17	−0.5 (4)	C30—C31—C32—C33	178.9 (2)
C15—C16—C17—C18	1.3 (4)	C31—C32—C33—C34	179.6 (2)
C16—C17—C18—C19	177.8 (2)	C37—C32—C33—C34	−0.7 (4)
C16—C17—C18—C13	−0.8 (3)	C32—C33—C34—C35	0.5 (4)
C12—C13—C18—C19	−0.4 (3)	C33—C34—C35—C36	−0.4 (4)
C14—C13—C18—C19	−179.1 (2)	C34—C35—C36—C37	0.5 (4)
C12—C13—C18—C17	178.2 (2)	C31—C32—C37—C36	−179.5 (2)
C14—C13—C18—C17	−0.5 (3)	C33—C32—C37—C36	0.8 (3)
N1—C10—C19—C18	176.51 (19)	C31—C32—C37—C38	0.8 (3)
C11—C10—C19—C18	−2.6 (3)	C33—C32—C37—C38	−179.0 (2)
C17—C18—C19—C10	−176.4 (2)	C35—C36—C37—C32	−0.7 (3)
C13—C18—C19—C10	2.2 (3)	C35—C36—C37—C38	179.0 (2)
C29—N2—C20—O2	−4.7 (3)	N2—C29—C38—C37	−179.50 (19)
C29—N2—C20—C21	172.35 (19)	C30—C29—C38—C37	−0.6 (3)
O2—C20—C21—C22	66.2 (3)	C32—C37—C38—C29	−0.1 (3)
N2—C20—C21—C22	−110.9 (2)	C36—C37—C38—C29	−179.8 (2)

### Hydrogen-bond geometry (Å, °)

**Table:**

*D*—H···*A*	*D*—H	H···*A*	*D*···*A*	*D*—H···*A*
N1—H1···O2	0.86 (3)	2.09 (3)	2.932 (2)	167 (2)
N2—H2A···O1^i^	0.82 (2)	2.19 (3)	3.003 (2)	169 (2)

Symmetry codes: (i) *x*−1, *y*, *z*.
